# miR-513a-5p regulates radiosensitivity of osteosarcoma by targeting human apurinic/apyrimidinic endonuclease

**DOI:** 10.18632/oncotarget.11003

**Published:** 2016-08-02

**Authors:** Nan Dai, Yi Qing, Yanping Cun, Zhaoyang Zhong, Chongyi Li, Shiheng Zhang, Jinlu Shan, Xiao Yang, Xiaoyan Dai, Yi Cheng, He Xiao, Chengxiong Xu, Mengxia Li, Dong Wang

**Affiliations:** ^1^ Cancer Center, Research Institute of Surgery, Daping Hospital, Third Military Medical University, Chongqing, 400042, P.R. China; ^2^ Department of Neurosurgery, Wuhan General Hospital of Guangzhou Military Command, Wuhan, 430070, P.R. China

**Keywords:** miR-513a-5p, osteosarcoma, radioresistance, APE1

## Abstract

Radiotherapy in osteosarcoma patients is problematic due to radioresistance; therefore, understanding the mechanism of radioresistance is integral to providing effective radiotherapeutic regimens for osteosarcoma. We now report the activity of an miRNA, miR-513a-5p, in stimulating radiosensitivity of osteosarcoma cells *in vitro* and *in vivo*. MiR-513a-5p expression is decreased in osteosarcoma tissue from patients and cultured osteosarcoma cell lines. However, exogenous re-expression of this miRNA in osteosarcoma cell lines, including HOS, U2OS and 9901, can induce sensitization to ionizing radiation. We also confirm that miR-513a-5p suppresses APE1 expression, and that both the redox and DNA repair activity of APE1 were decreased in miR-513a-5p expressing cell lines. By suppressing APE1, miR-513a-5p induces the DNA damage response which stimulates apoptosis after irradiation. Our report establishes miR-513a-5p as a radiosensitizing miRNA and identifies its activity in the suppression of APE1, which could directly lead to radiosensitization.

## INTRODUCTION

Osteosarcoma is the most common histological type of primary bone malignancy and is most prevalent in children and adolescents. The improvement of diagnostic imaging technologies and the combination of neoadjuvant chemotherapy and radical surgical *en bloc* resection have dramatically improved five-year survival rates of osteosarcoma patients from 45% to ∼71% in the last 40 years. [1]. However, osteosarcoma is still one of the leading causes of death and disability in children and adolescents with approximately 30–35% of patients suffering from local recurrence or distant metastasis. While radiotherapy is a powerful therapeutic strategy for solid tumors, its use in osteosarcoma is controversial because of radioresistance [2, 3]. Thus, understanding the mechanism of radioresistance of osteosarcoma is not only a key to appreciate the intrinsic radioresistance of solid tumors but also important for improving the effectiveness of the radiotherapeutic regimen for osteosarcoma.

Ionizing radiation (IR), the major form of irradiation used in radiotherapy, causes oxidative DNA base damage by stimulating reactive oxygen species (ROS) through hydrolysis. The resulting DNA base lesions are usually close to each other and, if unrepaired, convert to DNA double strand breaks, the most lethal type of DNA damage. Oxidative DNA base lesions are repaired by DNA base excision repair (BER), a fundamental pathway in the dynamics of radiosensitivity [4, 5]. Human apurinic/apyrimidinic endonuclease/redox effector factor (APE1) is an essential BER enzyme accounting for ∼95% of AP endonuclease activity in human cells. APE1 is highly expressed (reviewed in [6]) and is associated with radioresistance in various types of cancers, including osteosarcoma, according to previous studies by our group and others [7–15]. The correlation between APE1 and radioresistance was initially appreciated in the study which introduced human APE1 protein into radiosensitive *E. coli* which restored the radioresistance [16]. This study indicated that the DNA repair activity, which is evolutionarily conserved, is required for cell survival after IR. Direct evidence supporting the activity of APE1 in the radioresistance of osteosarcoma was shown in the study that sensitized HOS cells to Co-60 γ-irradiation using RNAi against APE1 [7]). These studies suggested that APE1 could serve not only as a predictive marker for intrinsic radioresistance but also as a promising therapeutic target to reverse the radioresistance of osteosarcoma.

As an essential gene for cell survival following irradiation, APE1 is abundantly expressed in human cells, but knowledge about its regulation is minimal. MicroRNA (miRNA),a class of endogenous single stranded non-coding RNA, has been one of the most exciting yet unexpected discoveries in oncology. Naturally occurring miRNAs are very short RNA transcripts which never translate into a protein, but act by negatively regulating protein expression during cellular processes such as growth, development, and differentiation through binding to the 3′-untranslated region (3′-UTR) of target genes. While miRNA is considered to be an important inhibitor of gene expression, the miRNA-mediated inhibition of APE1 remains unclear. In previous studies, we employed a high-throughput miRNA microarray to define changes in the miRNA profile of the osteosarcoma cell line HOS after knockdown of APE1 [17]. We speculated that the increased miRNA could be due to the feedback loop between APE1 and its regulatory miRNA. We screened and further confirmed that seven miRNAs were increased in APE1 knockdown HOS cells, and among them, miR-513a-5p exhibited an approximately 3-fold increase. Although the biological functions and intracellular targets of miR-513a-5p remain to be determined, previous studies suggest that miR-513a-5p is associated with cellular sensitivity to etoposide- and cisplatin-containing chemotherapy in retinoblastoma and non-small cell lung cancer (NSCLC) [18, 19]. Additionally, the level of miR-513a-5p in osteosarcoma is reduced based on the data from cell lines and clinical samples. This suggests a possible link between decreased miR-513a-5p and the radioresistance of osteosarcoma [17, 20].

Based on these data, we hypothesized that miR-513a-5p decreases APE1 expression and thus increases the radiosensitivity of osteosarcoma. The results reported here revealed that miRNA is an important intracellular regulator of APE1 expression, suggesting that controlling this process could result in radiosensitization of osteosarcoma.

## RESULTS

### miR-513a-5p is decreased in osteosarcoma and inversely correlated to APE1 expression

**Figure 1:**
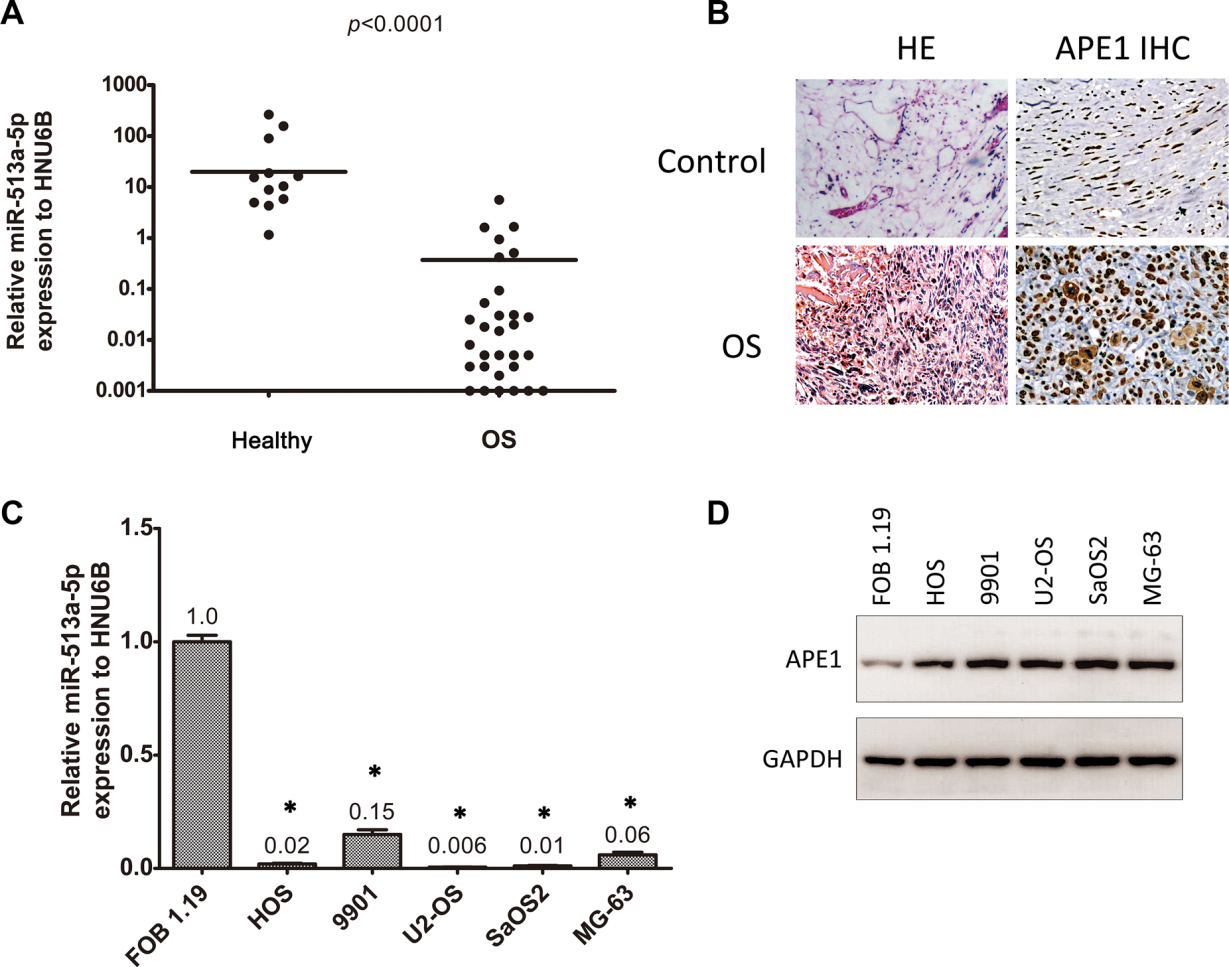
miR-513a-5p is decreased in osteosarcoma and inversely correlated to APE1 expression (**A**) The expression of miR-513a-5p in tissue from osteosarcoma patients (OS) and healthy donors was determined by quantitative RT-PCR and normalized with an internal control, HNU6B. The differences were statistically analyzed using one-way ANOVA. (**B**) The representative images of hematoxylin and eosin (HE) staining and APE1 immunohistochemistry (IHC) of FFPE tissue slides are from healthy donors (control) and osteosarcoma patients. (**C**) The expression of miR-513a-5p in human osteoblasts and osteosarcoma cell lines was determined by quantitative RT-PCR and shown in the bar graph. The APE1 protein levels from the same cell lines were detected by Western blot and representative blots are shown in (**D**). An asterisk (^*^) indicates that the difference between the marked group and the FOB1.19 group is statistically significant (*p* < 0.01).

We initially detected the expression of miR-513a-5p using quantitative PCR in formalin fixed paraffin embedded (FFPE) tissue from both osteosarcoma patients and healthy donors. The clinical characteristics of the cohort studied are displayed in Table 1. Reduced expression of miR-513a-5p is observed in bone tissue from both osteosarcoma patients and healthy donors compared to other miRNAs which have been reported to be highly expressed in osteosarcoma, such as let-7a or miR-451(20) (data not shown). As shown in Figure 1A, miR-513a-5p expression is significantly lower in samples from osteosarcoma patients than those from healthy donors (*p* < 0.0001). In addition, there is no difference among various histological types of osteosarcoma (*p* = 0.392). We further validated miR-513a-5p expression in established osteosarcoma cell lines and a normal osteoblast line. In agreement with the data from clinical samples, Figure 1C indicates that miR-513a-5p is decreased in osteosarcoma cell lines compared to the normal osteoblastic line FOB 1.19.

**Table 1:** The clinical characteristics of the cohort

Characteristics	Cases		Controls		*P* value
Age (Range)	18.5 (11–64)		21.5 (14–67)		0.569
Gender					0.689
Male	23	76.7	7	70.0	
Female	7	23.3	3	30.0	
Histology					
Osteoblastic	14	46.7	/		
Fibroblastic	7	23.3	/		
Chondroblastic	5	16.7	/		
Mixed	4	13.3	/		
APE1 Expression					
Negative	7	23.3	9	90.0	< 0.001
Positive	23	67.7	1	10.0	

As shown previously, miR513a-5p is increased in APE1 knockdown osteosarcoma cell lines, so we speculated that there could be an inverse correlation between miR-513a-5p and APE1. Therefore, we investigated the APE1 protein level in the same cohort of FFPE samples using IHC. The representative images for APE1 staining are shown in Figure 1B. We observed an inverse correlation between APE1 protein level and miR-513a-5p expression throughout the clinical samples we studied (r = −0.493, *p* = 0.001). In addition, APE1 protein levels were examined in osteosarcoma cell lines and normal osteoblasts (Figure 1D). The results demonstrated that the protein level of APE1 is elevated in osteosarcoma cells which further confirmed an inverse relationship with miR-513a-5p expression.

### Varying levels of miR-513a-5p alter radiosensitivity of osteosarcoma cells

**Figure 2:**
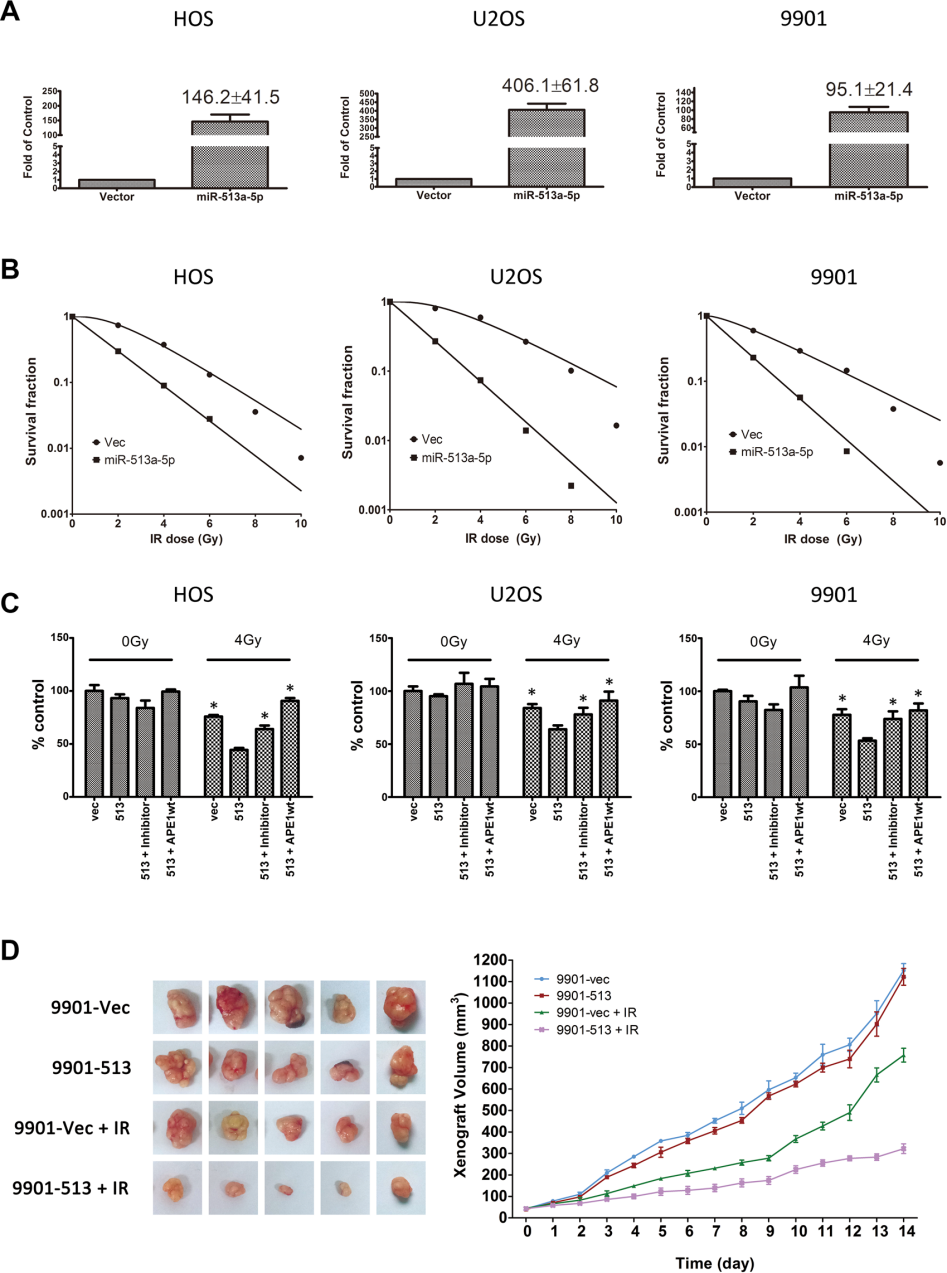
Increasing miR-513a-5p levels induced radiosensitivity of osteosarcoma cells (**A**) The stable expression of miR-513a-5p in osteosarcoma cells and control cells was determined by quantitative RT-PCR and shown in the bar graph. The fold increase is shown at the top of the bar of the miR-513a-5p group. The increase of miR-513a-5p in all three cell lines was statistically significant (*p* < 0.001). (**B**) The radiosensitivity of miR-513a-5p expressing HOS, U2OS and 9901 cells was determined by colony formation after 2, 4, 6, 8 and 10 Gy of X-ray irradiation and the detailed D_0_ and D_q_ values are shown in Table 2. (**C**) The overall survival of control (vec), stably-expressed miR-513a-5p cells (513), stably-expressed miR-513a-5p cells treated with miR-513a-5p inhibitors (513+inhibitor) and stably-expressed miR-513a-5p cells transfected with wildtype APE1 expressing vector (513+APE1wt) after 4 Gy X-ray or sham irradiation (0 Gy) were measured by CCK-8 assay. An asterisk (^*^) indicates that the difference between the marked group and the 513 group is statistically significant (*p* < 0.01). (**D**) The tumor growth curve of 9901-vec and 9901-513 cell xenografts post 4 Gy X-ray or sham irradiation is shown. The difference between 9901-vec+IR and 9901-513+IR is significant (*p* < 0.01).

Based on the studies of miR-513a-5p in osteosarcoma, we hypothesized that the downregulation of miR-513a-5p in osteosarcoma cells could be associated with the intrinsic radioresistance of osteosarcoma which has been reported to be due to the high expression of certain DNA repair proteins, including APE1. We performed *in vitro* experiments to correlate miR-513a-5p expression in osteosarcoma cells to radioresistance. We increased miR-513a-5p expression in osteosarcoma cells using a lentivirus expressing miR-513a-5p with a puromycin selection marker. An enzyme-resistant miR-513a-5p inhibitor was introduced to increase miR-513a-5p. Three osteosarcoma cell lines, including HOS, U2OS and 9901, with stable miR-513a-5p expression were measured for radiosensitivity using a colony formation assay. The exogenous miR-513a-5p was over-expressed 146-fold, 406-fold and 95-fold in HOS-513, U2-OS-513 and 9901-513 cells, respectively. As shown in Figure 2A, osteosarcoma cell lines with stable miR513a-5p expression were significantly more sensitive to IR when compared to the vector-expressing controls (all *p* < 0.01). The D_0_ and D_q_ values of each group are listed in Table 2. In addition to the radiosensitizing effect, stable expression of miR-513a-5p resulted in slightly impaired cell viabilities in all three cell lines as evidenced by reduced plating efficiency (PE) from 42% (HOS), 64% (U2OS) and 53% (9901) to 22%, 45% and 44%, respectively. We also performed an MTT assay to determine the overall cell viabilities of different osteosarcoma cell lines transiently transfected with either an miR-513a-5p mimic or inhibitor following 4 Gy irradiation (Figure 2C). We observed the same trend of radiosensitization by the miR-513a-5p mimic as determined by cells stably expressing miR-513a-5p using a colony formation assay (Figure 2B). On the other hand, despite the low expression profile of miR-513a-5p in all three osteosarcoma cell lines, radioresistance following IR was observed in the miR-513a-5p inhibitor-transfected osteosarcoma cell lines.

**Table 2:** D_0_ and D_q_ values of miR-513a-5p expressed osteosarcoma cell lines

	HOS		U2OS		9901	
	Vec	miR-513a-5p	Vec	miR-513a-5p	Vec	miR-513a-5p
**D_0_**	1.980198	1.637733	2.49004	1.489203	2.417795	1.382361
**D_q_**	4.071287	0.026204	5.871514	0.061057	1.428917	−0.0311

We further verified the radiosensitizing effects of miR-513a-5p *in vivo*. We injected 9901-513 and 9901-vec cells to the axillary fossae of each mouse. The volume of xenografts was measured daily after the xenografts became visible. When the volume of the xenograft reached 40 mm^3^ (∼5 days post inoculation, marked as day 0 in Figure 2C), the tumor-bearing mice received irradiation of 5Gy/fraction, for 3 fractions, within a 72 h-interval. The tumor growth curve showed that the mean size of the xenograft derived from 9901-513 cells after radiation was significantly smaller than that from 9901-vec cells (Figure 2D). Notably, in contrast with the *in vitro* cell viability results, the growth of xenografts derived from 9901-513 cells was similar to 9901-vec cells without irradiation, suggesting that miR-513a-5p exerts more complex effects on tumor growth *in vivo*. These results provide evidence for the radiosensitizing activity of miR-513a-5p in osteosarcoma both *in vitro* and *in vivo*.

### APE1 expression is suppressed by miR-513a-5p

**Figure 3:**
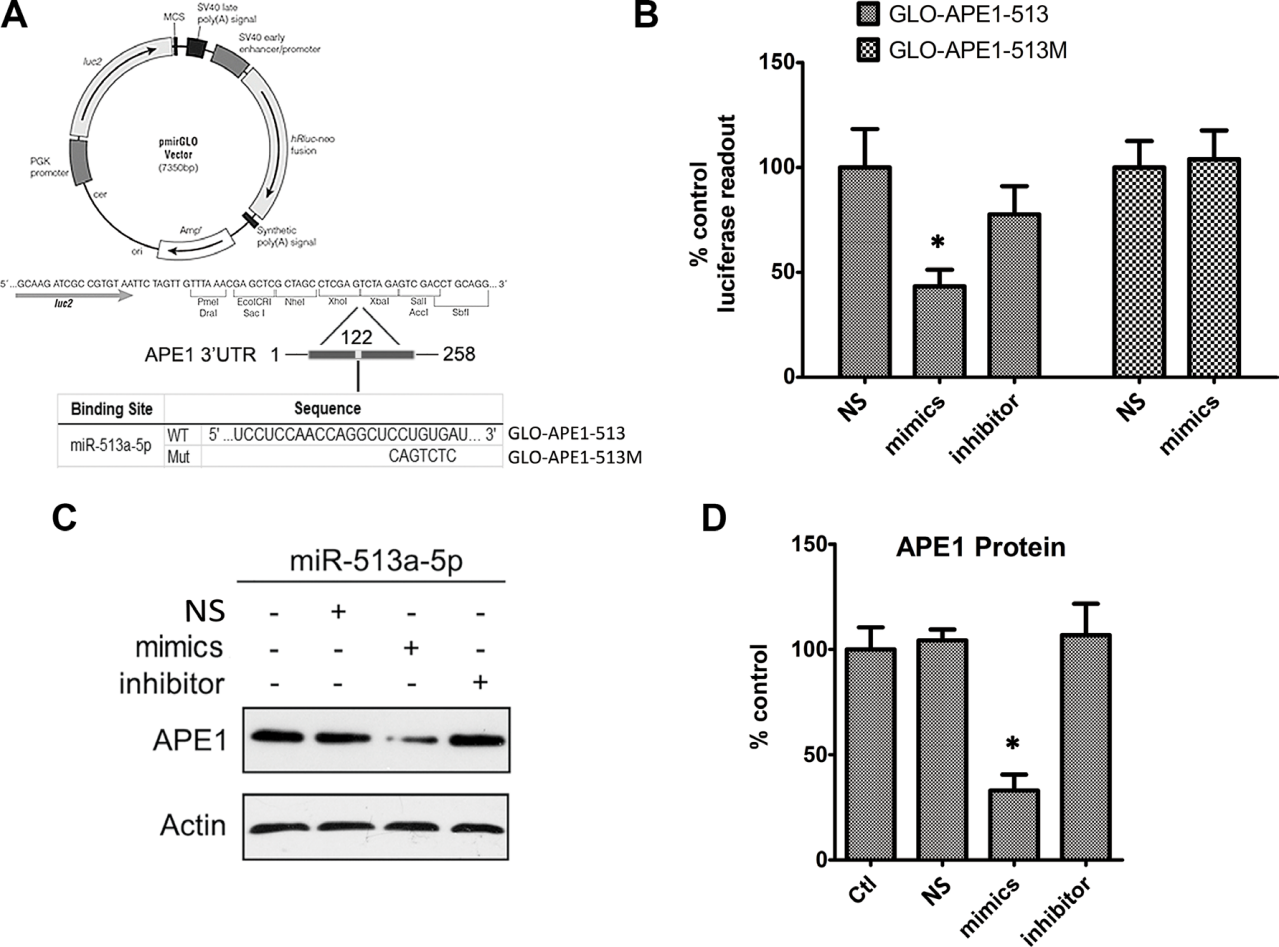
APE1 expression is inhibited by miR-513a-5p (**A**) The graph depicts the construction strategy of GLO-APE1-513 (WT) and GLO-APE1-513M (MT). The APE1-3′UTR sequence (258 bp) containing the putative miR-513a-5p binding site and mutant sites was synthesized and inserted respectively into the pmirGLO Dual-Luciferase miRNA target expression vector at XhoI and Xbal restriction enzyme sites. (**B**) The effects of miR-513a-5p binding of the 3′UTR of APE1 on the luciferase activity. HOS cells were transfected with GLO-APE1-513 vector and GLO-APE1-513M vector. Luciferase activity was detected at 48∼72 h after transfection with or without scrambled mimics, miR-513a-5p mimics and miR-513a-5p inhibitor, respectively. An asterisk (^*^) indicates that the difference between the marked group and the scramble-transfected group is statistically significant (*p* < 0.01). (**C**) APE1 protein expression was detected by Western blot after transfection with scrambled mimics, miR-513a-5p mimics and miR-513a-5p inhibitor in HOS cells. β-actin was used as the internal control. Both representative blots and statistical analyses (**D**) are shown. An asterisk (^*^) indicates that the difference between the marked group and the scramble-transfected group is statistically significant (*p* < 0.01).

Considering APE1’s suppression of radiosensitivity and the inverse relationship between APE1 and miR-513a-5p, we assumed APE1 could be an important intracellular target of miR-513a-5p in radiosensitization. To explore this mechanism, we initially attempted to confirm whether APE1 expression is decreased by miR-513a-5p. Combined with the algorithms in the TargetScan 6.2 database (http://www.targetscan.org/), we found that the putative binding region and key binding base pairs for miR-513a-5p are at the 3’UTR of APE1 (Figure 3A). We cloned a segment from the 3’ UTR of APE1 (258bp) which contains the putative binding region for miR-513a-5p, with either wildtype sequence or a sequence with the key bases mutated, to a commercially available luciferase reporter vector, pmirGLO-dual, downstream of the luciferase reporter gene (named GLO-APE1-513 and GLO-APE1-513M, respectively) (Figure 3A). We co-transfected the miR-513a-5p mimic with the GLO-APE1-513 reporter vector in HOS cells and observed that the luciferase activity at 48 to 72 h post transfection significantly decreased in miR-513a-5p mimic transfected HOS cells compared with the scrambled mimic group. When transfected with the GLO-APE1-513M reporter vector, the miR-513a-5p mimic did not decrease the luciferase activity suggesting that the mutated sites are critical to miR-513a-5p binding (Figure 3B). To link the luciferase results to APE1 transcription regulation both APE1 mRNA and protein expression in HOS cells were measured. We observed a significantly decreased APE1 protein level, but not mRNA level in the miR-513a-5p mimic transfected HOS cells, whereas increased APE1 expression was observed after transfection with the miR-513a-5p inhibitor (Figure 3C). The same experiments were performed in U2-OS and 9901 cells and similar results were observed (data not shown). Taken together, these results further confirmed that APE1 expression is decreased by miR-513a-5p.

### miR-513a-5p is correlated with intracellular APE1 levels post irradiation

**Figure 4:**
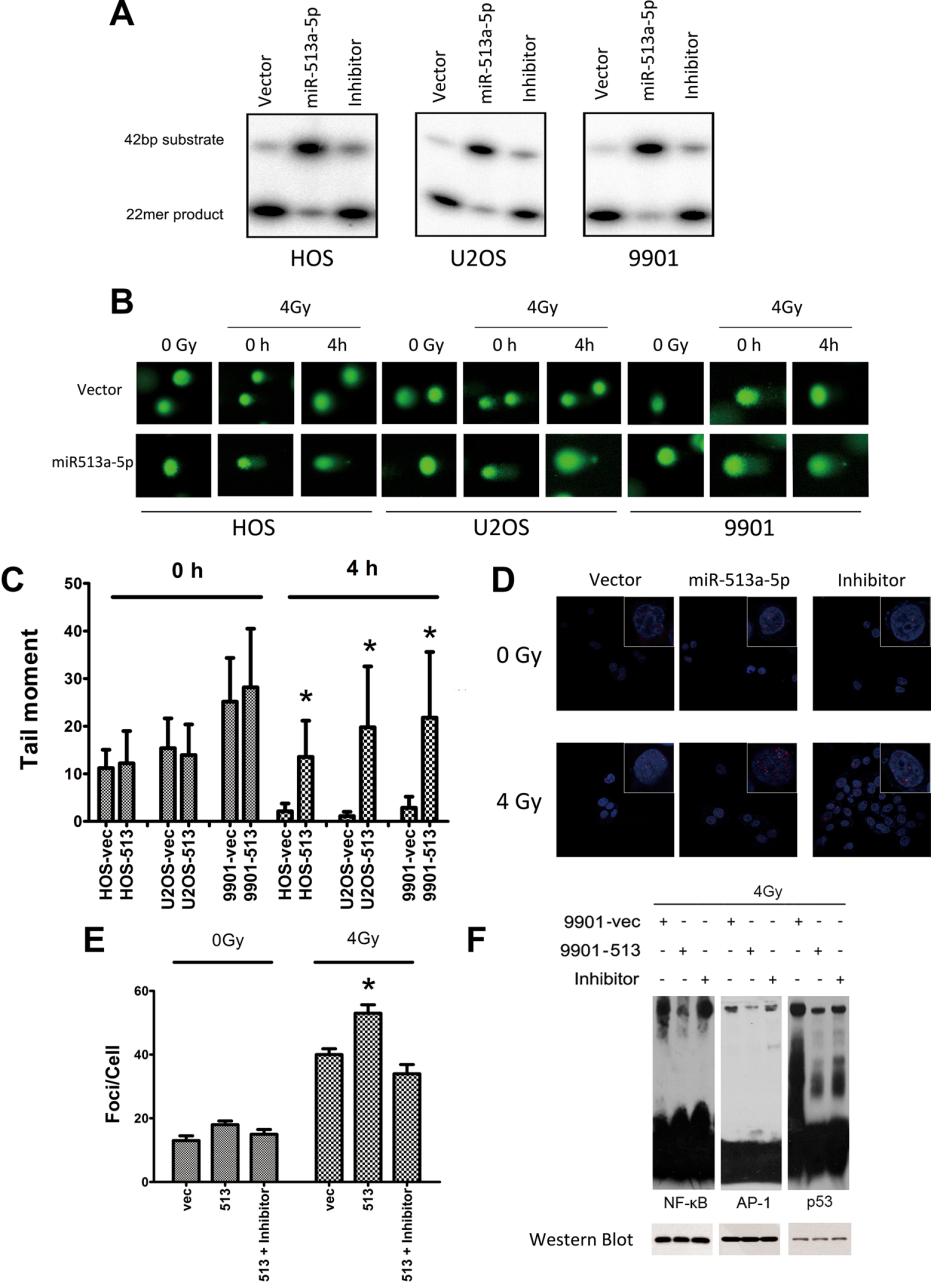
miR-513a-5p decreases DNA repair and redox activity of APE1 in osteosarcoma cells (**A**) AP endonuclease activities of APE1 protein in the control cell line, stably-expressing miR-513a-5p cells, and inhibitor treated HOS, U2OS and 9901 cells were detected by abasic site incision assay. (**B**) Osteosarcoma cells with stable miR-513a-5p expression and control cells received 4 Gy X-ray or sham (0Gy) irradiation, and were detected by alkaline comet assay immediately (0 h) or after recovery for 4 hours (4 h) to evaluate the overall DNA repair activity of single strand breaks. Representative images of the comet assays are shown. (**C**) Statistical analysis of comet assay and asterisk (^*^) indicates that the difference between the marked group and the vector-transfected group is statistically significant (*p* < 0.01). (**D**) γ-H2AX foci were visualized (red) at 4 hours post irradiation or sham by immunofluorescent assay. Nuclei were stained by DAPI as shown in blue. Only the representative images from each group are shown in the merged pattern. (**E**) Statistical analysis of γ-H2AX foci staining counts and asterisk (^*^) indicates that the difference between the marked group and the vector-transfected group is statistically significant (*p* < 0.01). The impact of miR-513a-5p level on the redox activity of APE1 was analyzed by EMSA using the probes containing the binding sequences of NF-κB, p53 and AP-1, which are transcription factors regulated by APE1 redox activity (**F**). The cells were harvested at 24 hour after 4 Gy X-ray irradiation. To verify the loading of nuclear extracts in each reaction was equal, Western blot against NF-κB (p50), p53 and AP-1 were shown in the bottom panel.

**Figure 5:**
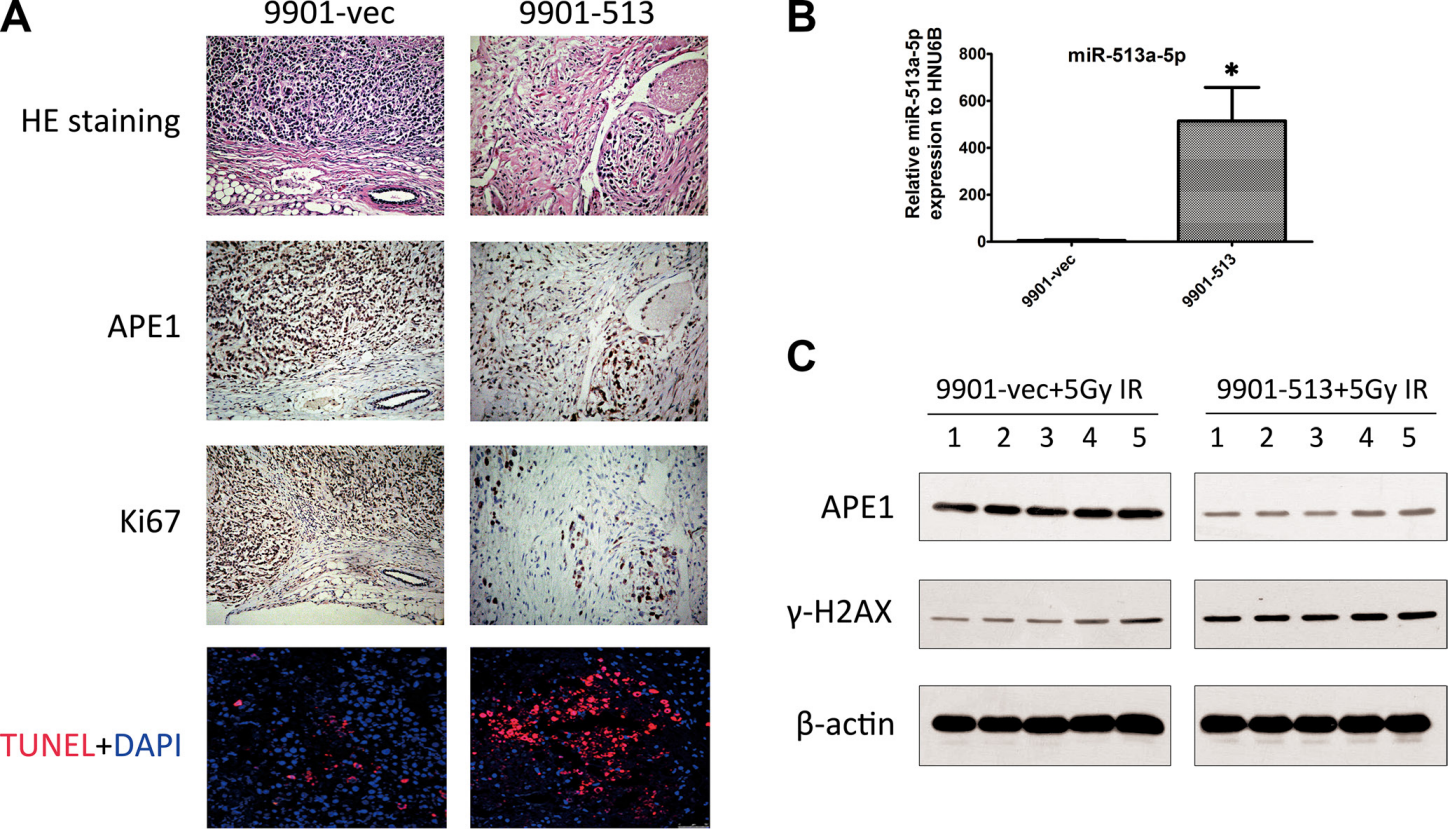
Effects of increased miR-513a-5p on xenograft radiosensitivity (**A**) HE staining, APE1 immunohistochemistry, TUNEL and Ki-67 staining of 9901-vec and 9901-513 xenografts after 5 Gy of X-ray radiation. Ki-67 is a marker for cell proliferation *in situ*. TUNEL assays were carried out using the commercial kit and protocol indicated in the Materials and Methods section. Apoptotic cells were stained by red fluorescence and all the nuclei were stained by DAPI shown as blue fluorescence. (**B**) The expression of miR- 513a-5p in 9901-vec and the 9901-513 xenografts was analyzed by quantitative PCR and the increase of miR-513a-5p in all three cell lines was statistically significant (*p* < 0.001). (**C**) The expression of APE1 and induction of γ-H_2_AX in each xenograft post 5 Gy irradiation of both groups was determined by Western blot, and the representative blots are shown.

To link the radiosensitizing effects of miR-513a-5p with the biological function of APE1, we examined whether APE1 activities correlate with the intracellular miR-513a-5p level. As shown in Figure 4A, AP endonuclease activity was significantly decreased in HOS-513, U2-OS-513 and 9901-513 cells after irradiation, whereas it was increased significantly after miR-513a-5p inhibitor transfection. The DNA base lesions and single strand break (SSB) DNA lesions were analyzed by comet assay subsequently. Four Gy IR causes a significant number of DNA base lesions and SSBs which can be effectively repaired by all three vector-expressing osteosarcoma cell lines, while these lesions were left unrepaired or repaired more slowly in miR-513a-5p-expressing cells (Figure 4B and 4C), suggesting that the AP endonuclease activity of APE1, together with the BER and SSBR capacity, were impaired in stably-expressing miR-513a-5p osteosarcoma cells. The DNA damage response marker Υ-H2AX was significantly increased in miR-513a-5p expressing 9901 cells while miR-513a-5p inhibitor transfection restored the genome integrity (Figure 4D and 4E). These data further confirmed that miR-513a-5p impairs DNA repair capacity in osteosarcoma cells and increases the DNA damage response. We also determined if an increase in miR-513a-5p could alter the redox activity of APE1 and the DNA-binding activities of NF-κB, p53 and AP-1, which are transcription factors reported to be increased by APE1 redox activity. As determined by EMSA at 24 h post irradiation, we found the amount of DNA binding complexes of NF-KB, p53 or AP-1 were significantly inhibited in osteosarcoma cells with increased miR-513a-5p, whereas the bound complexes were restored in miR-513a-5p inhibitor transfected cells (Figure 4F). In addition to these *in vitro* observations, we investigated the expression of APE1 protein in 9901-513 and 9901-vec xenografts by immunohistochemistry and Western blot. In the 9901-vec+IR group, intensive nuclear and cytoplasmic immunostaining of APE1 was observed in tumor cells, and the tumors in the 9901-513+IR group showed significant decreases in both nuclear and cytoplasmic immunostaining of APE1 and significant increases in miR-513a-5p expression (Figure 5B–5D). To determine the effect of miR-513a-5p on radiosensitivity of osteosarcoma *in vivo*, apoptosis and proliferation were evaluated by TUNEL and Ki-67 staining. We found more apoptosis and less proliferation in the 9901-513 group after radiation (Figure 5B). Moreover, γ-H2AX levels, which represent the DNA damage response, were elevated in xenografts with increased levels of miR-513a-5p (Figure 5D). These findings established an inverse correlation between radiosensitization by miR-513a-5p and the intracellular level of APE1.

### Radiosensitizing effect of miR-513a-5p is increased after suppressing APE1

**Figure 6:**
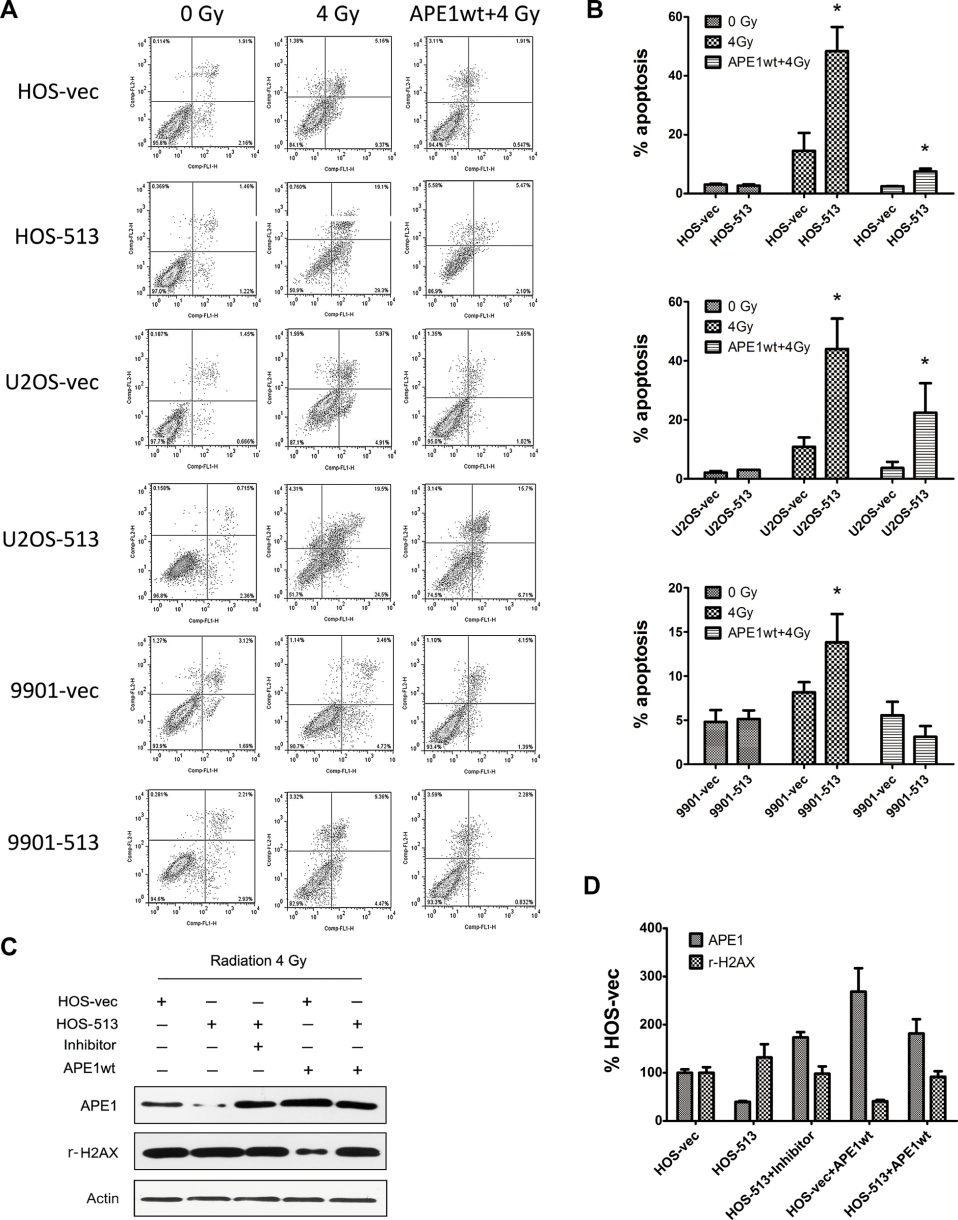
The radiosensitizing effect of miR-513a-5p is increased by suppressing APE1 Osteosarcoma cells with miR-513a-5p expression and control cells were first transfected with wildtype APE1 expression vector and irradiated with 4 Gy X-ray. The apoptosis of these cells was analyzed by flow cytometry after AnnexinV/PI staining. The representative flow cytometry is shown in (**A**) and the statistical results are shown in the bar graph in (**B**). An asterisk (^*^) indicates that the difference between the marked group and its vector transfected counterpart is statistically significant (*p* < 0.01). (**C**) The expression of APE1 and induction of γ-H_2_AX in cells with the indicated treatment after 4 Gy irradiation was determined by Western blot, and the representative blots and statistical results are shown in (**D**).

To confirm that the radiosensitizing effect of miR-513a-5p results from decreased APE1 levels, we introduced either an APE1 overexpression or knockdown lentivirus to increase and decrease APE1 levels. APE1 knockdown has been reported to cause cell death thus we employed AnnexinV/PI staining as well as an MTT assay to monitor cell death through apoptosis. Based on the results displayed in Figure 2A, total cell viability was significantly reduced in stably-expressing miR-513a-5p cells following IR. In contrast, we observed restored cell viability by exogenously expressing APE1 Figure 6A. In agreement with the *in vivo* data, the *in vitro* apoptosis assay (a representative flow cytometry graph is displayed in Figure 6A and the analysis in 6B) showed that miR-513a-5p stably-expressing osteosarcoma cells harbored more apoptotic cells at 24 hours following 4 Gy IR. Transfecting an APE1-overexpressing lentivirus in which the 3′UTR is absent makes the exogenous APE1 expression independent of control by miR-513a-5p, and in this case we observed that the apoptotic population was significantly reduced after IR (Figure 6C). Additionally, exogenously expressed APE1 significantly prevented DNA damage response activation, which was verified by reduced γ-H2AX levels. Thus, APE1 expression rescued the apoptotic phenotype, which was further confirmed by detecting PARP-1 cleavage by Western blot (Figure 6D).

## DISCUSSION

APE1, a gene essential to life in mammals, problematically increases radioresistance of osteosarcoma. The controlled suppression of APE by miRNA holds promising potential to overcome this problem and is a phenomenon that remains unexplored. We now report miR-513a-5p as the first validated miRNA for APE1. MiR-513a-5p, minimally expressed in osteosarcoma, is a radiosensitizing miRNA that decreases the expression of APE1, previously reported as a radioresistance gene. We increased or decreased APE1 expression utilizing miR-513a-5p in osteosarcoma cells, and thereby provided evidence that miR-513a-5p can increase radiosensitization through decreasing APE1 expression.

APE1 is abundantly expressed in the nucleus of mammalian cells throughout all the tissue types. In addition, in human cancer cells, APE1 expression is significantly increased, with an abnormal subcellular distribution. The expression of APE1 has been extensively explored but mostly at the posttranscriptional modification or protein levels. In contrast, the transcriptional expression of APE1 remains unclear. A few studies analyzed the promoter of APE1 and found that p53 and other transcription factors, as well as APE1 itself, could alter APE1 expression. It is known that transcriptional suppression by miRNA is an important mechanism at the 3′UTR of genes. Accumulated evidence shows that the mRNA levels of some genes are under the combined regulatory cascade of transcription factors and miRNA [21]. We propose a mechanism of APE1 gene suppression by miRNA. Future studies are required to correlate miR-513a-5p and APE1 expression in different tissues. If a significant reverse correlation can consistently be found, then this correlation may be universally conserved suggesting a significant cellular function.

Intriguingly, it has been previously reported that B7-H1, also known as Programmed Death-Ligand 1 (PD-L1), is the regulatory target for miR-513a-5p as well. PD-L1 has been extensively studied for its immunosuppressive signaling and as a potential target for cancer immunotherapy. Although previous studies of miR-513a-5p demonstrate suppression of PD-L1 in the context of an etoposide-induced cellular response [18], the overall evidence supporting the activity of PD-L1 in tumor chemo- or radiosensitivity is scarce. In contrast, considering its pivotal activity in DNA repair and transcription factor regulation the newly reported suppression of APE1 by miR-513a-5p provides a more plausible explanation to its radiosensitizing, or chemosensitizing, effects. In addition, our recent clinicopathological correlation study of PD-L1 and APE1 expression in gastric cancer demonstrated a positive correlation of PD-L1 and APE1 protein expression in cancerous tissue (Qing et al. in press), suggesting that this positive correlation could be due to regulation by a common miRNA regulator, miR-513a-5p. Further studies are necessary in order to elucidate the mechanism of the co-alteration of PD-L1 and APE1 expression and the pathological implications in tumorigenesis or tumor immunity. The crosstalk of APE1, a DNA repair protein, with PD-L1 needs to be explored in order to increase our understanding of tumor immunity.

The discovery of miRNA regulation of APE1 gene expression provides a viable strategy for future APE1 interference in cancer therapeutics. As an endogenous, short, single-stranded RNA, miRNA is quite safe and stable when transfected into human cells. The specificity of miRNA is the biggest concern as there are usually several intracellular regulatory targets for each miRNA. However, used in the appropriate context, this strategy has the promise to provide a more effective treatment for osteosarcoma.

To our knowledge, this is the first report on the miRNA suppression of APE1 gene expression and the establishment of the activity of miR-513a-5p in increasing radiosensitivity in osteosarcoma. We confirm that APE1 can be decreased at its 3′UTR by miRNA in osteosarcoma cells. A pan-cancer analysis should be initiated to discover additional potential miRNA control of APE1 expression in different cancer tissues. Understanding these control mechanisms may result in an effective radiosensitizing strategy in cancer therapy.

## MATERIALS AND METHODS

### Cell culture, human tissue samples and animals

293FT cells and human osteosarcoma cell lines HOS and SaOS2 were purchased from Cell Culture Center, Institute of Basic Medical Sciences, CAMS and PUMC, Beijing, China. U2-OS and 9901 were kind gifts from Professor Qing-Yu Fan [22, 23]. Human fetal osteoblast cell line hFOB 1.19 and MG-63 were kind gifts from Professor Yue Zhou. hFOB 1.19 cells were cultured as described previously [24]. Thirty OS paraffin-embedded samples were collected from patients who underwent OS resection or puncture biopsy without prior chemotherapy and radiotherapy in Daping Hospital and Xinqiao Hospital from 2010 to 2013. Nine healthy control samples were obtained from to-be-discarded bone fragments from similarly consented patients undergoing debridement surgeries for acute, traumatic injuries to the long bones. This study was approved by the Ethics and Research Committee of the Daping and Xinqiao faculty of Medicine, Third Military Medical University and written informed consent was obtained from all patients and healthy controls. All mice used were maintained and handled under institutional and regulatory guidelines and surgical procedures. Immunohistochemistry using sections from paraffin-embedded tumors were performed as described previously [22].

### Plasmid construction and transfection

The full length sequence of APE1-3′UTR (258 bp, microRNA.org database) containing the target sites for miR-513a-5p was synthesized and inserted into the pmirGLO Dual-Luciferase miRNA Target Expression Vector (Promega, USA) at XhoI and Xbal restriction enzyme sites. The mutated vector, GLO-APE1-513M, was constructed by replacing the seven bases at positions 135-141 of the APE1-3′UTR with scrambled bases (showed in Figure 2A). Production of lentiviral vector expressing hsa-miR-513a: A 506 bp genomic fragment containing hsa-miR-513a was cloned into lentiviral vector pLentis-CMV-GFP-MCS-PGK-PURO. The lentiviral particles were produced as described previously [25].

### Luciferase gene reporter assays

For transfection, HOS, U2-OS and 9901 cells were plated in 96-well plates for 12 h. At 48 h post transfection using Lipofectamine™ 2000, the cells were harvested. The intracellular luciferase activities were determined using the Dual Luciferase Reporter Assay kit (Promega, USA) according to the manufacturer’s protocol.

### Irradiation and colony formation assay

For irradiation experiments, the cell suspensions were counted and irradiated using an 8-MV X-ray with Elekta Precise Linear Accelerator (Stockholm, Sweden) [12, 26]. After irradiation, the cells were immediately aliquoted into individual wells of different plates and cultured for 48 to 72 h for the subsequent experiments. The colony formation assay was performed as described previously [27].

### AP endonuclease activity assay

The AP endonuclease activities of APE1 were evaluated by a well-characterized oligonucleotide cleavage assay as previously described [28].

### Comet assay

In order to detect the effects of miR-513a-5p on the level of DNA damage and repair in cells, the single cell gel electrophoresis assay was performed. The assay was carried out using the Comet Assay kit (Trevigen, Gaithersburg, MD, USA) according to the procedure of Yang *et al.* [29].

### Electrophoretic mobility-shift assay

Electrophoretic mobility shift assay (EMSA) was accomplished with the LightShift Chemiluminescent EMSA kit (Pierce, USA) according to the method described by Li *et al.* with minor modifications [30]. EMSA was accomplished according to the user’s instruction with minor modifications. Briefly, 5 μg of nuclear extracts were incubated with 3′-biotin labeled double-stranded probes. The probes containing NF-κB consensus, AP-1 consensus and p53 consensus and their complementary strands were purchased from Beyotime Institute of Biotechnology. After incubation, samples were separated on a pre-run 5% polyacrylamide gel at 100 V for 90 min and transferred to Zeta-Probe GT nylon membrane (Bio-Rad). The probes were detected by HRP-conjugated streptavidin (1:300) and the bands were visualized by ECL kit reagents. The resultant bands were quantified using the imaging software Quantity One (Bio-Rad).

### Cell proliferation assay

Cell proliferation was detected using the cell counting kit-8 (CCK-8, Beyotime Inst Biotech, China) according to manufacturer’s instructions.

### Apoptosis

Apoptosis assays were performed with the FITC-conjugated AnnexinV antibody/propidium iodide (PI) staining kit (Ferment, Canada) according to the manufacturer’s indicated protocol. The samples were analyzed by flow cytometry in the Third Military Medical University flow cytometry facility.

### Western blot analysis and antibodies

Western blots were performed following the previous protocol reported by our group [30]. The monoclonal antibody against hAPE1 was from Novus Biological (Littleton, CO).

### Quantitative RT-PCR and RT-PCR

Total RNA from HOS, U2-OS and 9901 cells was isolated by using a Trizol reagent (Invitrogen, USA) following the manufacturer’s protocol. The U6 gene was used for normalizing each sample. Subsequently, quantitative RT-PCR was performed in triplicate using SYBR Premix Ex Taq II (Takara, Japan) and the Bulge-Loop™ miRNA qPCR Primer Set (RiboBio, China) in a LightCycler 480 Real-Time PCR System (Roche, USA). Primer pairs for hsa-miR-513a-5p and U6 were purchased from RiboBio Co. Ltd (China). Primer pairs for APE1 and β-actin were designed to yield 120 and 289 bp separately as described previously [30, 31].

### *In vivo* experiments

Cell suspensions of 9901-513 and 9901-vec cells (1 × 10^7^) were injected subcutaneously to both axilla of each nude mouse: 10 mice in total and 10 tumors for each cell type. When the xenograft tumors grew to approximately 40 mm^3^ on day 5 after cell inoculation, 10 tumor-bearing mice were randomized into the radiation group and non-radiation group (5 mice per group). The radiation group was irradiated on the tumor area with 5 Gy of X-ray, 3 times at 72 h intervals. On day 14, xenografts from each group were isolated and tumor volumes were determined. Tumor sizes were measured before each treatment and after the mice were sacrificed and calculated according to the formula: tumor size (mm^3^) = (maximum diameters ×minimum diameters ^2^)/2. An inhibition ratio was calculated after the mice were sacrificed by the formula [1-(treated tumor average volume/untreated tumor average volume)]×100%.

### *In situ* apoptosis detection by TUNEL staining

The formalin-fixed and paraffin-embedded 5 mm-thick sections of all tumor samples were analyzed for apoptosis by terminal dUTP nick end labeling (TUNEL) staining using the Apoptag Kit (Intergen, Purchase, NY, USA).

### Statistical analysis

All data was expressed as the mean ± S.D. Statistical analysis of data was assessed using the Student’s *t-*test and 1-way ANOVA with the software SPSS 13.0. Differences with *p* < 0.05 were considered statistically significant.
